# Recent advancement in bioeffect, metabolism, stability, and delivery systems of apigenin, a natural flavonoid compound: challenges and perspectives

**DOI:** 10.3389/fnut.2023.1221227

**Published:** 2023-07-26

**Authors:** Peng Chen, Fuchao Chen, ZhiLei Guo, Jiexin Lei, Benhong Zhou

**Affiliations:** ^1^Department of Pharmacy, Renmin Hospital of Wuhan University, Wuhan, Hubei, China; ^2^Department of Pharmacy, Sinopharm Dongfeng General Hospital, Hubei University of Medicine, Shiyan, Hubei, China; ^3^Department of Pharmacy, Wuhan Fourth Hospital, Wuhan, Hubei, China; ^4^Department of Endocrinology, Renmin Hospital of Wuhan University, Wuhan, Hubei, China

**Keywords:** apigenin, biological activity, bioavailability, drug delivery, natural flavonoid

## Abstract

Apigenin is a bioflavonoid compound that is widely present in dietary plant foods and possesses biological activities that protect against immune, cardiovascular, and neurodegenerative diseases and cancer. Therefore, apigenin is widely used in food and medicine, and increasing attention has been drawn to developing new delivery systems for apigenin. This review highlights the biological effects, metabolism, stability, and bioactivity of apigenin. In addition, we summarized advancements in the delivery of apigenin, which provides some references for its widespread use in food and medicine. Better stability of apigenin may enhance digestion and absorption and provide health benefits. Constructing delivery systems (such as emulsions, nanostructured lipid carriers, hydrogels, and liposomes) for apigenin is an effective strategy to improve its bioavailability, but more animal and cell experiments are needed to verify these findings. Developing apigenin delivery systems for food commercialization is still challenging, and further research is needed to promote their in-depth development and utilization.

## 1. Introduction

**Figure 1:**
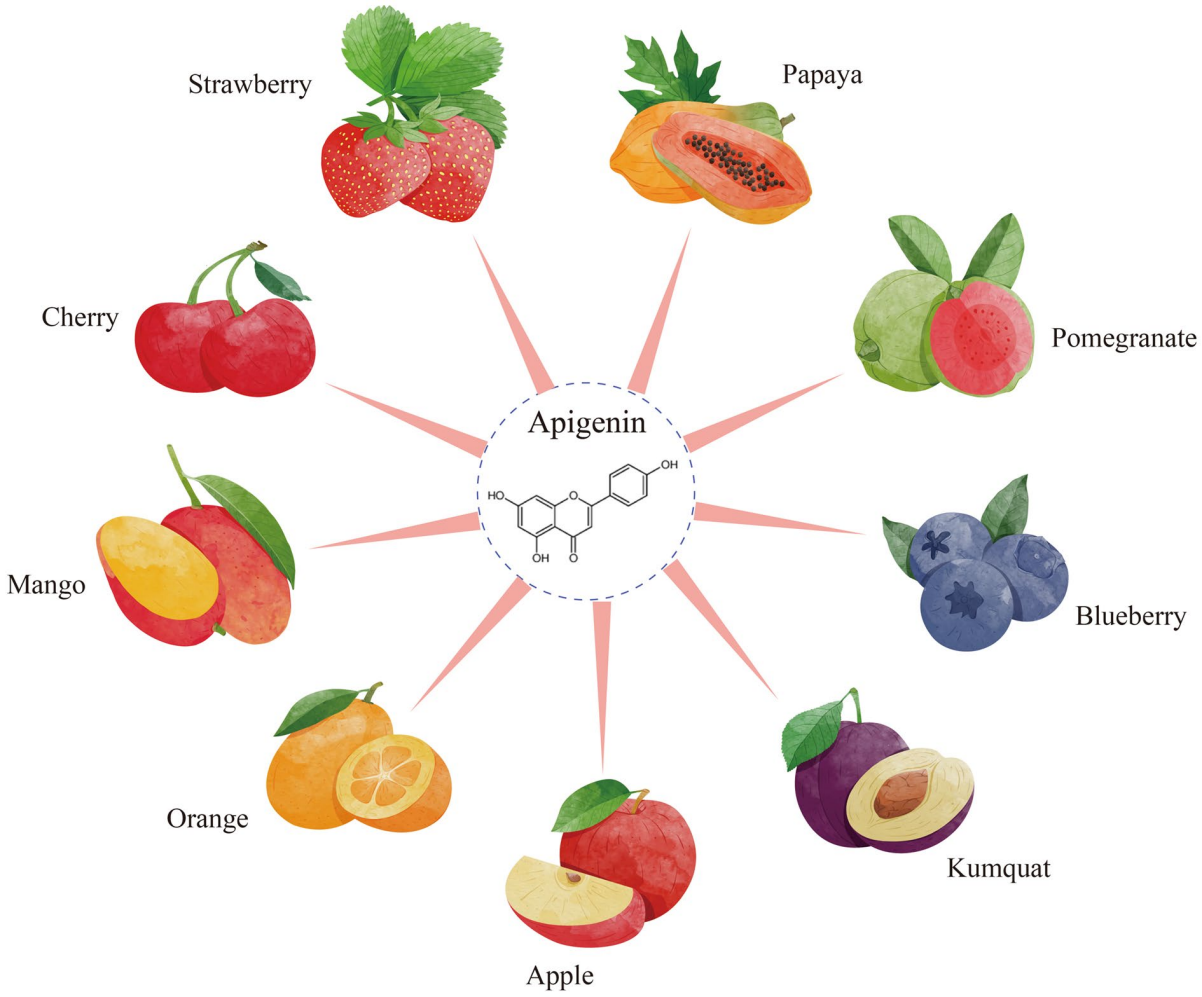
The resources of apigenin from fruits.

Apigenin, 4′,5,7-trihydroxyflavone, which is abundant in vegetables such as parsley, celery, and onions and in fruits such as strawberries and oranges (Figure 1) that have been historically used as medicinal plants worldwide (1). It is a yellow crystalline compound, its chemical structure comprises multiple hydroxyl groups, namely, in the C-5 and C-7 positions of ring A and the C-4 position of ring B. Because of its increased permeability and diminished water solubility, the plasma membrane of the host organism is readily penetrated by this substance (2). Apigenin is lipophilic and can be deactivated in the acidic environment of the gastrointestinal tract, leading to lower bioavailability, which limits its potential use in healthcare products and functional foods (3).

Nevertheless, because of its lipid-soluble properties, apigenin can be used as a natural pigment in food processing. It has been confirmed to possess a wide range of biological and therapeutic activities, including antioxidant, anti-inflammatory, anti-cancer, anti-genotoxic, antiallergic, neuroprotective, cardioprotective, and antimicrobial effects (4). As a nutrient, apigenin has many applications in health products, medicine, cosmetics, and many others (5). Therefore, it is crucial to develop methods for enhancing apigenin bioavailability.

As previously reported, apigenin bioavailability can be significantly improved by constructing apigenin delivery systems (6). In this review, the extraction of apigenin is discussed and its biological activity, digestive absorption, and stability are also discussed. Furthermore, we review the latest advances in apigenin delivery systems to offer valuable insights into the future development and application of apigenin.

## 2. Apigenin biosynthesis in plants

Naturally, apigenin is biosynthesized by phenylpropanoid metabolic pathway where 4-coumaroyl-CoA compound is produced from phenylalanine amino acid through the shikimate pathway in plants (7). In the presence of chalcone synthase, a crucial enzyme in the route of flavonoid biosynthesis, the molecule 4-coumaroyl-CoA interacts with malonyl-CoA to create chalconaringenin, the backbone of flavonoids (8). Chalconaringenin is the main intermediate chalconoid that undergoes spontaneous cyclization into a compound called naringenin by an isomerase called chalcone (9). The enzyme flavone synthase catalyzes the conversion of naringenin to apigenin by forming a single double bond between the C2-C3 atoms of the ring C. For the C-or O-glycosylation, hydroxylation, and methylation of apigenin to create its derivatives, other enzymes, specifically glycosyltransferases, methyltransferases, and hydroxyltransferases, are required (10). Lee H. and colleagues have discussed the use of *Escherichia coli* for the production of apigenin and its derivative, genkwanin, from the tyrosine molecule (11). Marin and colleagues demonstrated the *de novo* synthesis of apigenin utilizing the microorganisms *Streptomyces albus* (12). Chemically, the first trustworthy apigenin synthesis method was proposed by Hutchins and Wheele, and it included the creation of chalcone (III), bromination of chalcone (III), demethylation, and debromination (13).

## 3. Extraction of apigenin

Apigenin possesses strong physiological and biochemical effects; therefore, the extraction and purification of apigenin from natural resources are very important for designing and developing apigenin products with higher bioactivities. Commonly used extraction methods in the food industry include pressurized solvent, enzymatic, heat reflux, soxhlet, supercritical fluid, ultrasound-assisted, and microwave-assisted extraction (14–25). The principles of apigenin extraction, advantages and disadvantages of various methods, and research achievements are summarized in Table 1.

**Table 1:** The principle, advantages and disadvantages of different extraction technology of apigenin.

Extraction method	Extraction principle	Examples	Advantages	Disadvantages	Reference
Organic solvent extraction	Apigenin has high solubility in different organic solvents.	Apigenin has high solubility in different organic solvents.	Mature technology, low equipment cost, simple operation and easy industrialization.	The extraction rate is low, the extraction time is long, the amount of solvent is large, difficult to recover, a variety of organic solvents make the safety risks greatly increased.	(14–19)
Supercritical fluid extraction	A solute in the mixture to be separated in a certain supercritical region near the critical point.	The extraction rate of apigenin was 61.80 μg/g under the condition: 25°C, 8 ~ 14 MPa, 125 s.	High extraction efficiency, high yield, high purity, low energy consumption, shortened extraction time, lower equipment price, cheaper extractant, non-toxic, suitable for recovery.	Higher equipment costs.	(20, 21)
Enzymatic hydrolysis assisted extraction	Specific enzymes are used to degrade or destroy endogenous pectin, glycoprotein, cellulose, etc. In cell walls, cell molds, reduce mass transfer resistance, and promote the dissolution of active ingredients.	Under the condition of enzyme reaction temperature 40°C, time 30 min; and a concentration of 0.4 mg/mL at pH 5.5，the yield of apigenin was 25.3 mg/g.	Mild extraction conditions, favorable for maintaining the activity of apigenin, improving extraction efficiency, improving product purity, and having great development prospects and application potential.	Industrial enzyme preparations are costly.	(22)
Ultrasonic assisted extraction	The multi-stage effects, including cavitation, mechanical, and the thermal effect, increase the frequency and velocity of movement of the extracted components	The recovery rate of apigenin ranging from 72.7 to 89.5% under the conditions by using 1.0 mol/L 1-butyl-3-methylimidazolium methylsulfate ([Bmim][MS]) aqueous solution at pH 1.0 as the extraction solvent; 200 W for 90 min (solid: liquid ratio was 1: 10).	High extraction efficiency, low energy consumption, shortened extraction time, lower equipment price and simple operation.	The equipment is still at the laboratory level, and has not been industrialized.	(23)
Microwave assisted extraction	Under the action of microwave electric field, many substances composed of polar molecules will strongly oscillate, causing rapid generation of a large amount of heat energy, leading to cell rupture	The extraction rate of apigenin was 104 μg/g, the yield was significantly improved compared with conventional extraction method.	High extraction efficiency, low solvent consumption, and high extraction rat.	Microwave has a selective temperature effect on polar substances, so it is required to have a small dielectric constant.	(24)
Ultrasonic-microwave synergistic extraction	Combining ultrasonic vibration and microwave heating to enhance the extraction efficiency	The apigenin yield was over 80%.	Overcome ultrasonic vibration, low noise, large noise, and uneven microwave penetration.	The mass transfer mechanism of ultrasonic-microwave synergistic extraction and the effect on the structure and activity of apigenin are still unclear.	(25)

Although there are many methods for extracting natural apigenin, their economic cost is high, and degradation or isomerization of apigenin often occurs during the extraction process (26). Existing evidence shows that there are a series of isomers and impurities in extracted apigenin; thus, its safety and usability in food processing and is extremely restricted. Microbial production and development technologies using fungi, algae, and yeast that meet the strict requirements of additives in the fields of food, pharmaceuticals, and livestock have gained increasing attention (27). This method has gained high praise in the food processing field because of its low cost, low energy consumption, and pollution-free nature; however, this technology has not matured and has not reached the scale of commercial mass production. To improve the efficiency of apigenin extraction and product quality, the scientific development of industrial production can be promoted by combining and exploiting beneficial characteristics of various extraction methods.

## 4. Bioactivity of apigenin

### 4.1. Antioxidant activity

Apigenin is an antioxidant that quenches singlet oxygen and scavenges peroxyl radicals (28). After the body absorbs oxygen, the oxygen can quickly interact with anions to form superoxide anion radicals, which are then converted into free radicals, including hydroxyl (OH•), superoxide (O2•^−^), nitric oxide (NO•), nitrogen dioxide (NO2•), peroxyl (ROO•), and lipid peroxyl (LOO•) (29, 30). An excess of free radicals and oxidants give rise to a phenomenon known as oxidative stress (OS), a deleterious process that can lead to cell damage and endogenous dysfunction (31). Long-term OS can accelerate aging and lead to several chronic disorders. In fact, many oxidants, such as hydrogen peroxide, nitrate, metal ion and glutamic acid, have been proven to induce cell dysfunction and diseases (32). Through literature research, large amounts of published studies have concentrated on the beneficial function of apigenin on OS-induced progressive diseases such as cancer, neurodegenerative diseases, cardiovascular disease, liver injury, and diabetes mellitus, both *in vitro* and *in vivo* (33). In general, apigenin can improve cell viability and/or alleviate tissue damage by increasing the resistance to oxidative stress inducers. It has been found that the key to the protective activity of apigenin is its ability to scavenge endogenous ROS and reduce malondialdehyde (MDA) levels. Further studies report, that apigenin reduced ROS and MDA levels, thereby enhancing antioxidant enzyme activities, such as those of superoxide dismutase (SOD), catalase, and glutathione peroxidase (GSH-Px), as well as the upregulation of antioxidant response proteins, such as nuclear factor erythroid 2-related factor 2 (Nrf2) and AMP-activated protein kinase (AMPK) (34, 35). Overall, apigenin can be considered a novel antioxidant that can decrease the risk of OS-induced disorders.

### 4.2. Anti-inflammatory activity

In recent *in vivo* and *in vitro* studies, there has been increasing interest in the anti-inflammatory activities of apigenin (36). In an *in vitro* study, apigenin prevented the injury response of lipopolysaccharide (LPS)-stimulated RAW 264.7 macrophage cells by enhancing the reduction of NO (37). In these processes, apigenin decreased the levels of pro-inflammatory cytokines TNF-α, IL-18, and IL-6, and downregulated the expression of enzymes (COX-2 and iNOS), as well as reducing the intracellular ROS production. It has been shown that apigenin can inhibit the activity of intracellular cell adhesion molecules (ICAMS), monocyte inflammatory protein (MIP-1α), and monocyte chemotactic protein (MCP-1α) inhibitors induced by LPS in an *in vivo* study of mouse leukocytes, resulting in an anti-inflammatory response (38). The downregulation of these pro-inflammatory factors by apigenin may be due to action of some transcriptional factors and kinases, such as extracellular signal-regulated kinases (ERK), NF-kB, and mitogen-activated protein kinase (MAPK) (39).

Glial cells, such as microglia and astrocytes, mediate neuroinflammation which is triggered by the activation of the innate immune system in the brain to cope with inflammation (40). Microglia and astrocytes can be activated by exogenous infection or irritation, releasing inflammatory cytokines to magnify neuroinflammation, leading to enhanced or prolonged brain pathology. Therefore, it is helpful to attenuate this inflammation to combat neurodegenerative diseases. The anti-inflammatory properties of apigenin were observed in BV2 microglia stimulated by LPS, as proven by the activation of GSK-3β/Nrf2 signaling pathway that attenuated the expression of IL-6, IL-1β, and TNF-α (41). Apigenin-induced transformation of IBA1-positive cells into the amoebic phenotype was observed in isolated rat microglial cultures and was related to an increase in the expression of the activated M1 spectral markers OX-42 and iNOS and decreased expression of the M2 spectral marker CD206 (42). Taken together, these results demonstrate that apigenin has anti-inflammatory and neuroprotective properties and could serve as a neuroimmunomodulatory agent (43).

### 4.3. Anti-cancer effects

Owing to the potent anti-proliferative effects of apigenin on different types of human cancer cells, including colon, bladder, breast, skin, prostate, and liver cancer cells, apigenin has a potentially broad application in cancer prevention and treatment (44, 45). A large number of experiments *in vitro* or *in vivo* have confirmed the biological effects of apigenin, showing that it has good anti-tumor activity (46).

Some possible mechanisms involved in the anti-cancer properties of apigenin include down-regulation of NF-κB pathway, inactivation of various kinases, and modulation of proteasomal degradation of the HER-2/neu proteins (47). It has been confirmed that apigenin is a selective protein kinase CK2 inhibitor, and evidenced by study results showing an increased apigenin-induced cell death rate in CK2α-high acute myeloid leukemia cells than that in CK2α-low acute myeloid leukemia cells (48). Furthermore, apigenin has been reported to trigger cell cycle arrest and promote and activate apoptosis in cancerous cells. Additionally, NF-κB was inactivated via apigenin inhibition of the Akt signaling-associated protein expression and p65 phosphorylation (49). Moreover, apigenin exerted chemopreventive effects on cancer cells by regulating the expression of antioxidant enzymes and the accumulation of ROS in lung cancer cells (50). Further studies have reported that apigenin triggers apoptosis via the tumor necrosis factor (TNF) receptor, activating ligand receptor (TRAIL-R)-mediated caspase-dependent cell death pathways in tumor cells (51). These findings suggest that combining apigenin with chemotherapeutic drugs may enhance cytotoxicity against cancer cells.

Ferroptosis is a new form of cell death described by Dixon et al. in 2012 and is characterized by glutathione consumption and lipid peroxide accumulation (52). An increase in endoplasmic reticulum stress, suppression of the cystine/glutamate antiporter, and activation of mitogen-activated protein kinases (MAPK) and mitochondrial voltage-dependent anion channels contribute to this process (53). An increasing number of studies have shown that ferroptosis has a highly complex relationship with cancer and could be an innovative treatment option for cancer (54). Consequently, it is necessary to conduct clinical trials of ferroptosis-inducing medicines for cancer treatment. Interestingly, several studies have reported that apigenin induces ferroptosis and kills tumor cells (33). According to Adham et al., the treatment of the multiple myeloma cell line NCI-H929 with apigenin resulted in ferroptosis, autophagy, apoptosis, and cell cycle arrest. However, apigenin cytotoxicity was completely ameliorated by the ferroptosis inhibitor, ferrostatin-1 (55). In a study by Liu et al., mesoporous magnetic nanosystems were developed for the delivery of apigenin, and it was found that the typical characteristics of ferroptosis included cellular lipid peroxidation levels and ROS levels in A549 cells were significantly increased using the targeted apigenin-loaded Fe2O3/Fe3O4@mSiO2-HA nanocomposite delivery system and the underlying mechanisms were mainly upregulation of ferroptosis associated genes COX2 and p53 and downregulation of GPX4 and FTH1 (56).

In addition, apigenin has anti-allergy ability, regulates blood lipids, prevents cardiovascular diseases, and can be used as a natural pigment in the food industry (57–59).

## 5. Digestive absorption, metabolism, and transport of apigenin

Owing to the important biological and pharmacological activities of apigenin, pharmacokinetic testing has been fully exploited to study its absorption, metabolism, distribution, and excretion. These findings are particularly beneficial for evaluating the optimal dose of apigenin for disease prevention and treatment.

### 5.1. Digestion and absorption of apigenin

The bioactivity of apigenin primarily originates from the release from raw food materials. To our knowledge, in food and herbal sources, the active apigenin is found in the form of various sugar moieties. During digestion, apigenin glycosides survive in the stomach through acid hydrolysis and enter the duodenum unchanged (4). Therefore, the location and degree of glycosylation affect apigenin absorption in the gastrointestinal tract (60). The distribution of the enzymes needed to generate bioactive apigenin and the characteristics of the linked sugar moiety affect the next step of digestion and absorption. Different cells metabolize apigenin intracellularly via enzymes present in the brush border epithelium (61). However, nondigestible glycosides require extracellular deglycosylation, which can be performed by bacteria and their associated enzymes that exist in the colon. Microbial alpha-glucosidase can facilitate the digestion of apigenin which is attached to rhamnose, whereas human beta-glucosidase cannot (60). Hanske et al. studied the deglycosylation of apigenin-7-glucoside by human intestinal microorganisms in rats and found that it promoted the biological activity of apigenin to a great extent (62).

It has been demonstrated that 5–10% of apigenin can be absorbed after the consumption of polyphenols (63). The absorption of apigenin is mainly mediated by the gastrointestinal tract (GI) prior to its entry into the bloodstream and liver. A study using a rat intestinal irrigation model showed that apigenin was immediately absorbed by the intestine after aglycone apigenin administration (64). Remarkably, there are various absorption routes for apigenin in different parts of the intestine. In the jejunum and duodenum, active and passive vehicle-mediated saturation mechanisms stimulate the absorption of apigenin, while in the ileum and colon absorption occurs via passive transport. However, reports on the absorption rate of apigenin are inconsistent (65). A study by Gradolatto et al. was consistent with the fact that apigenin is slowly absorbed and metabolized after oral administration, and even slowly eliminated (detected in blood circulation after 24 h) (66). However, another study conducted by Chen et al. reported a high absorption rate of apigenin after oral administration to rats (detected in blood circulation after 3.9 h) (67). A possible explanation for the difference in these findings is that the animal strains used in the two studies were different, the former using SD rats and the latter using Wistar rats (67, 68). It has been proved that animal strain difference is one of the main factors affecting the pharmacokinetic performance (68). Therefore, the absorption rate of apigenin requires further investigation.

### 5.2. Metabolism and transport

Growing evidence indicates that two main phases are involved in apigenin metabolism. In the liver, phase I metabolism of apigenin is accomplished by liver enzymes such as cytochrome P450, flavin-containing monooxygenase (FMO), and nicotinamide adenine nucleotide phosphate (NADPH) (60, 63). In phase II metabolism, the intestinal and hepatic circulation are involved in the biotransformation of apigenin (67). The phase II metabolism of apigenin mainly involves glucoaldehyde and sulfur acidification (60). In the process of metabolism, the principal biochemical metabolites of apigenin are luteolin as well as sulfated and glucuronic acid conjugates (19). Apigenin is excreted in the urine and feces, with higher concentrations in urine (69). Based on a literature review, it is clear that the age and sex of rats are important factors affecting apigenin excretion (66). Briefly, immature male and female rats, like mature female rats, excreted a higher percentage of the mono-glucuronoconjugate of apigenin than the mono-sulfoconjugate of apigenin (10.0–31.6% versus 2.0–3.6%, respectively). Mature male rats excreted the same compounds in an inverse ratio (4.9 and 13.9%, respectively). In addition, animal research evidence has shown that considering its slow metabolism and excretion, the accumulation of apigenin in the body is easy to understand.

**Figure 2:**
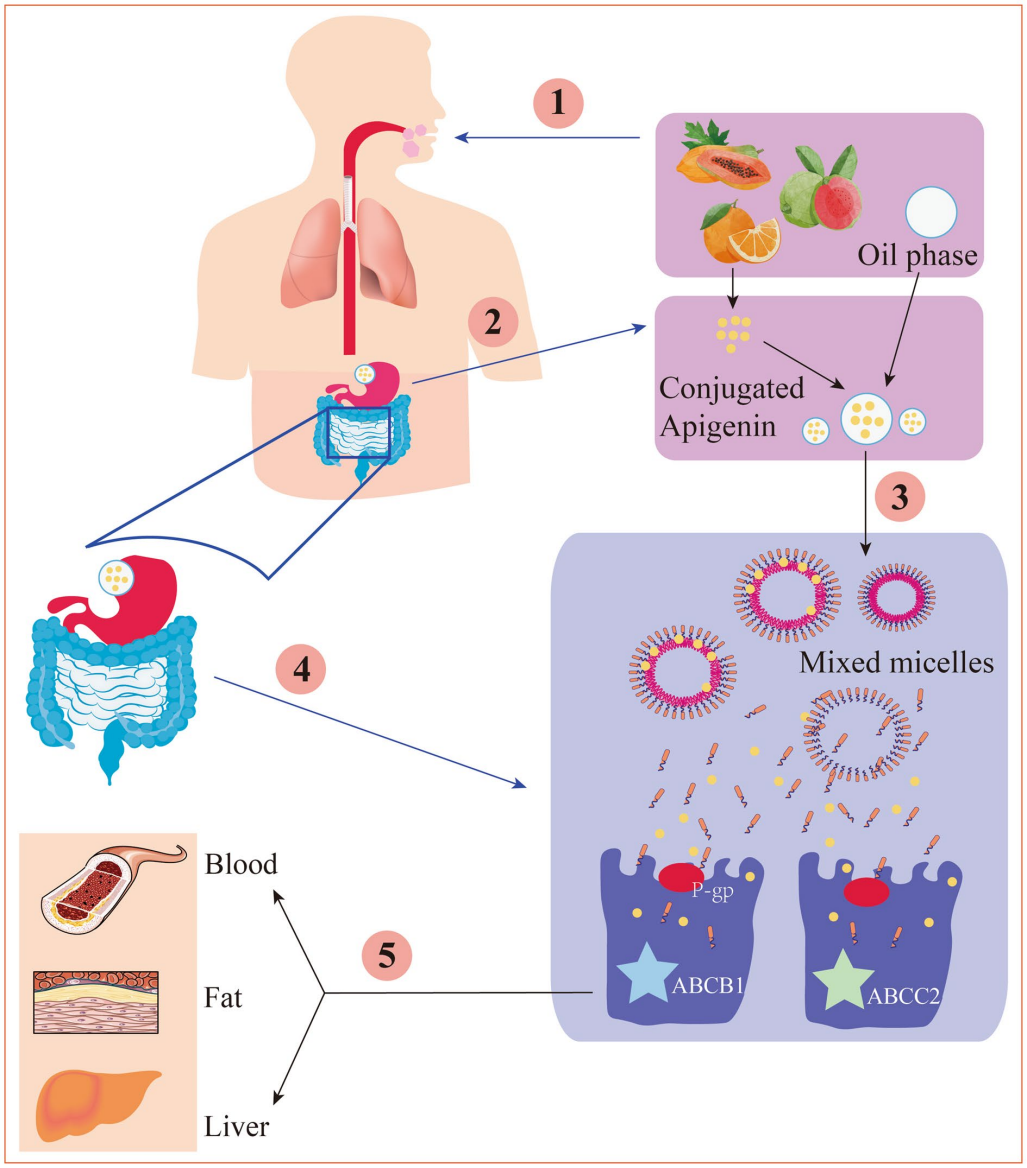
The digestion and absorption mechanism of apigenin in gastrointestinal tract. ① Under the action of chewing mechanical force, enzyme digestion and a small amount of oil, apigenin is released from the food substrate into the oral cavity. ② Apigenin is dissolved into the oil drips, then was hydrolyzed in the stomach. ③ Apigenin is released from the oil drips to form a mixed micella in the small intestine. ④ Through P-gp-mediated transport or other ways, the mixed micelles are absorbed by the epithelial cell layer. ⑤ Under the action of ABCB1 and ABCC2, the chylomicrons are transported to the lymph circulation and thereby enter the blood or physical organs.

After intestinal digestion and absorption, apigenin is converted into glucuronic acid conjugates and secreted back into the lumen of the gut, decreasing net absorption. Furthermore, conjugated apigenin may also be transported through the efflux transporters multidrug resistance protein-1 (also referred to as P-gp, ABCB1, and CD243) and multidrug resistance-associated protein-2 (also referred to as ABCC2 and CMOAT) (Figure 2), the abundances of which could be significantly changed under different disease states (70). Additionally, apigenin can be modified by methylation, sulfation, and glucuronidation, all of which affect its bioactivity and distribution (71).

### 5.3. Distribution of apigenin

It is generally accepted that people and animals cannot synthesize apigenin but can only obtain it from food. Indeed, apigenin can be synthesized by a variety of plants.

Human organs and tissues swiftly and equally distribute apigenin after it is absorbed by the body, and numerous studies have demonstrated the presence of apigenin in the serum, lung, kidney, brain, thyroid, ovary, womb, intestine, and liver. In addition, after apigenin intake by the body, it can also be found in the urine and feces (28). In particular, the blood and liver have been shown to have considerably higher apigenin content. Meyer et al. studied the relationship between dietary intake of apigenin and circulating levels in a range of healthy human volunteers (ages 21–41 years, mean body mass index 23.9 kg/m^2^) (72). The results showed that systemic levels of apigenin following the ingestion of apigenin-rich parsley (mean intake: 149.5 ± 35.2 g) peaked at about 7 h after oral intake, resulting in a mean apigenin serum concentration of 127.0 ± 24.3 nM (34.3 ± 6.57 ng/mL). The concentration of apigenin in tissues is determined by the expression and genetic variants of lipoprotein receptors and cholesterol carriers, which affect its accumulation in target organs (73).

### 5.4. Bioavailability of apigenin

The bioactivity of apigenin depends primarily on its bioavailability after digestion and absorption (74). Bioavailability is defined as the percentage of the provided chemical which can be absorbed and used for storage or physiological functions, and it is closely linked to bioaccessibility (75). Bioavailability refers to the conversion of apigenin from the food matrix to mixed micelles during digestion and rendering it available for intestinal absorption.

As a bioactive compound, the bioefficacy of apigenin is affected by many factors that include molecular structure, digestibility, food matrix, bioaccessibility, and transporter and metabolizing enzyme availability (76). Published studies have confirmed that apigenin has a low solubility in fat and water, with the solubility of 2.16 μg/mL and 0.001–1.63 mg/mL in water and non-polar solvents, respectively (77). Due to the low oral bioavailability of apigenin, its clinical application and promotion are limited. One of the core factors regulating the bioavailability of apigenin is its transformation in the intestinal mucosa to a large-molecular-weight glucuronic acid glycosidic conjugate. Thus, the net absorption of apigenin is severely reduced in the intestinal lumen (61). It has been reported that both the bioavailability and distribution of apigenin are also can be affected by formation of conjugates by methylation, sulfation, and glucuronidation. An intestinal epithelial metabolism model simulated by Caco-2 monolayers demonstrated that apigenin can be translated into a glucuronic acid conjugate by uridine 5′-diphosphate glucuronic acid transferase metabolism in the intestinal epithelium. It was further demonstrated that apigenin exhibited an apparent permeability coefficient (P_app_) ranging between 10 to 5 cm/s, suggesting that apigenin has high hydrophilicity and good intestinal absorption (78). UDP-glucuronic acid transferase (UGT) plays a significant role in accelerating apigenin metabolism rate of in the intestinal tract (79). Gut-secreted apigenin in Gunn rats increases the levels of the UGT1A isoform, enhancing its properties through hepatic anion efflux transporters for efficient metabolism and compensatory regulation of intestinal UGT2B, thereby limiting its bioavailability and increasing its disposition.

## 6. Stability and influence factors of apigenin

Many types of food can be converted from raw materials to final products for consumption or intermediate products for cooking and storage by processing techniques such as grinding, washing, drying, and heating. During processing, Fe and Cu ions can easily penetrate the food substrate because of frequent contact of the food with pipes and equipment containing these two metals on the surface. Additionally, dietary flavonoids can be affected by certain food treatments. The stability of apigenin is continuously damaged by Fe/Cu, especially at 37°C, and its stability decreases sharply (80). Further studies show that the bioactivity (apoptosis induction, intracellular ROS generation, DNA damage, and growth inhibition) of apigenin was significantly decreased after heat treatment with temperatures of 37°C and 100°C or Fe/Cu supplementation (80). As expected, the greatest reduction in the bioactivity of apigenin was observed after treatment with high temperatures, especially those above 100°C.

At present, only a few studies have evaluated the effects of heating processes on the phenolic composition of individual fruit and vegetable juices, such as kiwi, orange, and tomato. Generally, the results of these studies vary based on the available treatment, food matrix, and processing conditions. According to Sentandreu et al., the ordinary pasteurization of orange juice (90°C for 30 s) had little influence on its phenolic content (81). However, a study conducted by Morales-de la Pea found that immediately following processing, ultra pasteurized orange juice had higher concentrations of apigenin (5.32 ± 0.93 to 14.76 ± 1.28 mg/100 mL) than the control (7.20 ± 0.65 to 8.83 ± 3.44 mg/100 mL), and the enhanced content has not declined at the end of storage (82). Nevertheless, apigenin content was reduced in sweet potato leaves with increased blanching time due to leaching of constituents into water, or possible enzyme activity (83).

A study conducted by Hostetler revealed that apigenin concentrations increased four-fold (from 1 to 5 mg/g) after incubation with chickpea flour, flax seed, and almond for 20 h at 37°C (84). The highest stability of apigenin was observed at pH 3, whereas it gradually degraded at pH values ranging from 5 to 7. There was a higher content of apigenin-7-glucoside (3.0 ± 0.4 mg/g dry weight [dw]) in extracts from fresh chamomile compared to that in the dried sample (1.0 ± 0.3 to 2.0 ± 0.4 mg/g dw) (85). It has been reported that the content of apigenin in Kumquats (*Fortunella crassifolia*) exceeded 21 mg/100 g (fresh weight), but was less than 1 mg/100 g in *F. japonica* juice. In another study, vegetable snacks were prepared according to the ratio of parsley: carrot: onion: broccoli of 1:11.4:5.5:2.1 (86–88). The contents of apigenin in the dough and final products were 40.7 ± 4.9 and 38.3 ± 11.6 mg/100 g dry matter, respectively, showing that the apigenin content only reduced slightly during baking. The apparently higher apigenin content in snack products compared to the dough could be explained by differences in the distribution of phytochemicals in one or more batches of dough, or by improved flavonoid extractability during baking.

## 7. Advances in apigenin delivery systems

**Figure 3:**
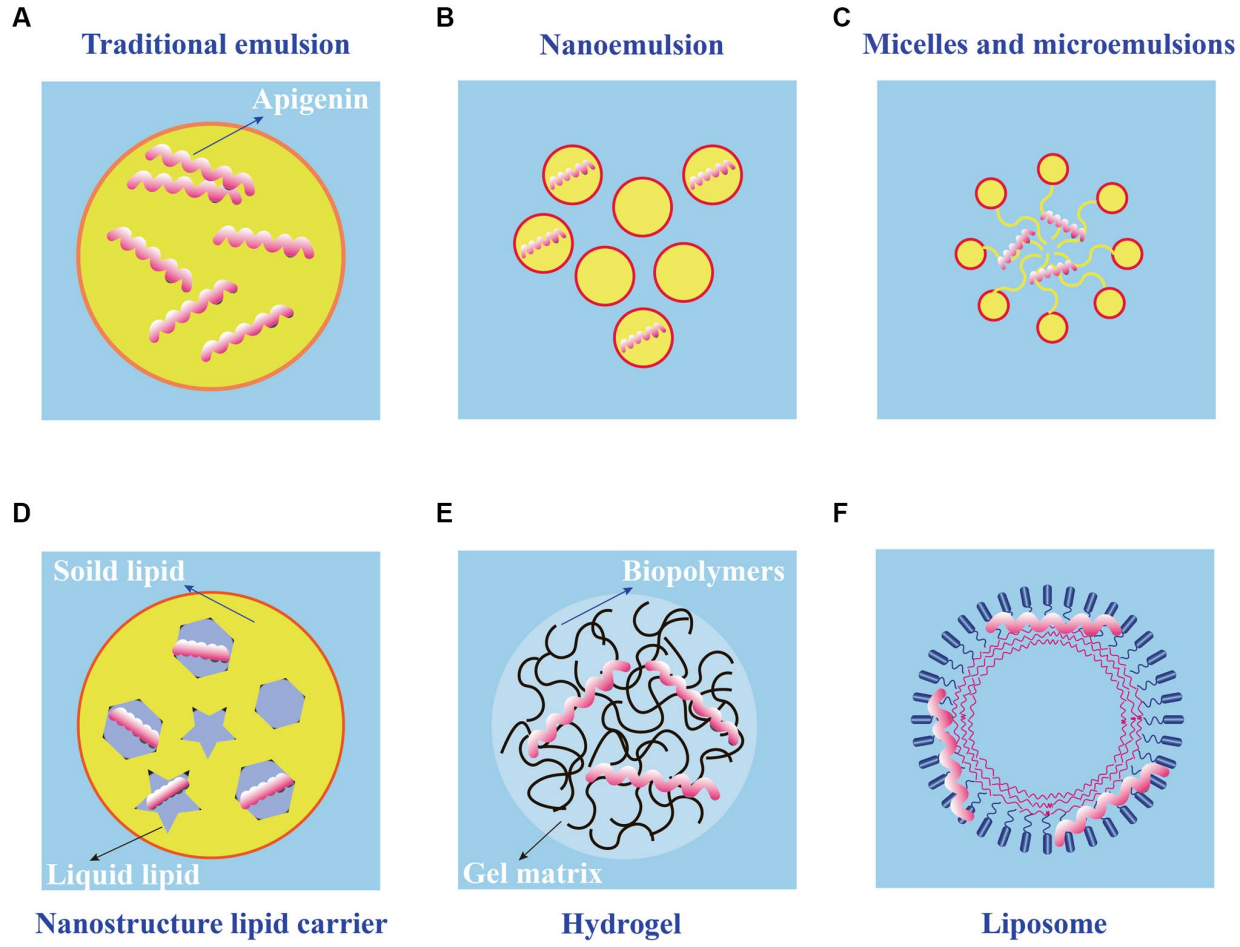
Schematic diagram of different types of apigenin delivery systems. **(A)** Traditional emulsion. **(B)** Nanoemulsion. **(C)** Micelles and microemulsions. **(D)** Nanostructure lipid carrier. **(E)** Hydrogel. **(F)** Liposome.

Application of apigenin as a nutraceutical is currently restricted in the food industry because of its low bioavailability, high chemical instability, and poor water solubility. To overcome these shortcomings, several studies have been conducted to develop different apigenin delivery systems. Various delivery systems are available including liposomes, hydrogels, nanostructured lipid carriers, microemulsions, nanoemulsions, and emulsions. The digestion and absorption of apigenin in the gastrointestinal tract are affected by the delivery system, which improves apigenin bioavailability and chemical stability, thereby enhancing its therapeutic effects (89). Various apigenin delivery systems are shown in Figure 3 and Table 2. The physical stability and water dispersibility of apigenin can be improved using a delivery system.

**Table 2:** Summary of report on the production of apigenin-based NPs, particle size and their importance in increasing bioavailability.

Delivery system	Advantages	Disadvantages	Application	Reference
Traditional emulsion	Enhance chemical stability, antioxidant activity and bioavailability of the active compounds.	Culation or aggregation (to a large extent); larger particles.	In energy drinks, dairy products; food ingredients.	(90–92)
Nanoemulsion	Low surfactant; dynamic stability; the bioavailability of the active compounds was significantly improved.	Flocculate or coalesce (to a lesser extent).	Drug delivery and targeted therapy.	(93, 94)
Micelles and microemulsions	Thermodynamic stability.	Recipitation; high level surfactant is required.	Energy drinks; natural colorant.	(95)
Nanostructure lipid carrier	Avoid organic solvents; improve the bioavailability of apigenin; change the transport mechanism of apigenin in biofilm.	Difficult in mass production and supply of raw materials.	Anticancer apigenin carriers, apigenin extracts substitute; lipophilic nutrition health food, functional food.	(96, 97)
Hydrogel	It can maintain a certain shape, absorb a lot of water; strong antibacterial activity; non-toxic and highly biocompatible; good release control ability of apigenin.	There is little research in the field of food.	May be used as packaging materials for foods with high water content.	(95, 98)
Liposome	High biocompatibility; improve targeting; improve the bioavailability and stability of apigenin.	The preparation cost is high and the production process is relatively complex.	Drug delivery and targeted therapy.	(99, 100)

### 7.1. Emulsions

The use of emulsions is a common approach to apigenin delivery. When two immiscible liquids are combined, the result is an emulsion fluid, in which one numerous tiny droplets of one liquid (droplet component or dispersed phase) are suspended in a continuous component (liquid phase) (101). Typical emulsions are of two main types: oil-in-water (O/W) and water-in-oil (W/O), both of which are potentially unstable because of their thermodynamic properties. For traditional emulsions (Figure 3A), bioavailability is increased, and the release of bioactive factors can be controlled; therefore, it has attracted the attention of many scholars and has become a research hotspot (90). The low solubility of apigenin may be resolved by encapsulation in oil-in-water (O/W) emulsions, resulting in the creation of new functional food products. Using high-pressure homogenization, Abcha et al. formulated and examined the physiochemical stability of apigenin-loaded food-grade O/W submicron emulsions (91). After one month of storage, the formed O/W emulsions maintained their physical stability with a minor decline in the PDI and dav values. In particular, the maximum retention rate (approximately 93%) was achieved by emulsions created at 100 megapascals (MPa) (92). These findings offer valuable insights for using high-pressure homogenization as a potentially effective method for the development of stable food-grade apigenin-loaded O/W emulsions, and for other nutraceutical formulations enhanced with hydrophobic flavonoids.

Compared with traditional emulsions, the nanoemulsions (Figure 3B) are smaller in particle size (usually 2–500 nm) and uniformly distributed (102). Nanoemulsions can resist the physical destabilization caused by gravitational separation, flocculation, and/or coalescence, and the water solubility and bioavailability is higher than traditional emulsions (103). Chou TH et al. prepared apigenin nanoemulsions using an anti-oxidative polymeric amphiphile, d-α-tocopheryl polyethylene glycol 1,000 succinate (TPGS), hydrogenated soy lecithin (HL), black soldier fly larvae (BSFL) oil, and avocado (AV) oil through pre-homogenization and ultrasonication method (93). The results found that apigenin nanoemulsions had higher chemical stability and antioxidant ability than that of apigenin emulsions. In addition, Jangdey MS et al. develop a potential novel formulation of carbopol-based nanoemulsion gel containing apigenin using tamarind gum emulsifier and the nanoemulsion formulation was stable for 3 months at 4°C or at ambient temperature (25°C) (94). More interestingly, the *ex vivo* skin permeation experiments revealed that the amount of apigenin permeated through skin from nanoemulsion gel (76%) was significantly higher than that from pure drug suspension and marketed product (49%). Therefore, the utilization of nanoemulsions is a good candidate for application of topical apigenin delivery.

Microemulsions (Figure 3C), which spontaneously develop from the coemulsifier, emulsifier, oil phase, and water phase at proper ratios, are clear, thermodynamically stable dispersion systems (104). Microemulsions have a uniform distribution and droplet sizes are much smaller (10–100 nm) in size than those in emulsions. The oil/water interfacial tension decreases to an extremely low level (10–3 mN/m) when the microemulsions are formed, further saturating both the water and oil phases by up to 70%. Zhao et al. prepared a microemulsion containing apigenin and a complex of hydroxypropyl-β-cyclodextrin (HP-β-CD). The resulting microemulsion increased apigenin release than that without HP-β-CD (95). Additionally, the antioxidant activity of the apigenin-loaded microemulsion was higher than that of the microemulsion without. Based on these observations, combining a microemulsion with an inclusion complex is a successful strategy for increasing the bioavailability of apigenin.

### 7.2. Nanostructured lipid carriers

Nanostructured lipid carriers (NLC) are a pharmaceutical colloidal delivery system combining the benefits and eliminating the defects of solid lipid nanoparticles (SLNs) and oil-in-water (O/W) nanoemulsions (105). The hydrophobic core of the particles in the NLCs is composed of a solidified fat phase with a loosely ordered structure that prevents morphological alterations and the escape of bioactive molecules. The structures of the NLCs are shown in Figure 3D. NLC nanoparticles are more hydrophilic than solid lipid nanoparticles (106). Lipophilic bioactive substances are more soluble in liquid lipids than in solid lipids. Thus, when the amount of liquid lipids increases, drug loading, encapsulation efficiency, and lattice defects also increase. The pharmacokinetics, stability, and adhesion of bioactive compounds are enhanced by NLCs because of good stability, high encapsulation efficiency, and high drug loading. Many studies have shown that the bioavailability, chemical stability, and dispersion of lipophilic materials increase significantly with the application of NLCs (107). Furthermore, the food safety, nutritional value, and function of bioactive materials were enhanced by NLCs; as a result, the release of encapsulated compounds was well controlled. The oral bioavailability of apigenin NLCs is approximately five times higher than that of free apigenin, and oral administration of this material has no adverse effects on the organs of experimental animals (108). Production and *in vitro* testing of apigenin-loaded NLCs by Ding et al. revealed that the ideal apigenin-NLC particle size was 46.1 nm (96). *In vitro* release showed that the optimal apigenin-NLCs exhibited a sustained release property compared to free apigenin. The NLCs displayed better bioavailability and penetrability than the drug extracts. Because apigenin is a lipophilic compound, the lipid core of NLCs can induce chylomicrons to carry apigenin to achieve transmembrane transport (97). The adsorption of NLCs to the gastrointestinal wall increases the bioavailability of apigenin by increasing the contact time of with intestinal epithelial cells. The above studies provide evidence for NLCs as appropriate carriers for apigenin. Further studies are needed to determine whether various molecular polarities would alter the sustained-release behavior of nanoparticles in NLCs and the bioavailability of apigenin in the human body.

### 7.3. Hydrogel

Using physical or chemical bonding to covalently connect hydrophilic polymers, hydrogels (Figure 3E) are a type of three-dimensional network that can hold several times their dry weight in water without dissolving (109). Filled hydrogels are emulsion-based delivery systems that shield biologically active materials from chemical degradation and digestion in storage and gastrointestinal environments. When used to maintain high-water meals or as food packaging, nanoemulsions or nanoparticles improve the antibacterial properties of hydrogels. According to the *in vitro* release kinetics, Zhao et al. reported that an O/W microemulsion in gellan gum hydrogels as a carrier of apigenin produced a Fickian diffusion-controlled mechanism for release under acidic conditions (95). The *in vitro* release under weakly alkaline conditions is an erosion-controlled mechanism. A further study demonstrated that apigenin-loaded hydrogels (HGs) was able to release 96.11% apigenin within one day and had an optimal hydrogel percent entrapment effectiveness of 87.15 ± 1.20 when made utilizing gellan gum-chitosans (GGCHs) (98). Additionally, in normal and diabetic wound tissues, apigenin GGCH-HGs have been confirmed to possess greater wound treatment and considerable antioxidant potential. These results suggest that hydrogels may be a potential release system for hydrophobic drugs when administered orally. However, research on the delivery of apigenin through hydrogels *in vivo* is limited. Creating apigenin hydrogel with the correct cross-linking agents can increase its stability and controlled-release capabilities in the digestive tract (110).

### 7.4. Liposomes

Liposomes consist of closed microvesicles with a bilayer of lipid molecules (Figure 3F) encapsulating the internal aqueous medium (111). They can decrease drug toxicity, improve the stability of active ingredients, and provide targeted and sustained-release benefits. Water molecules and liposomes can deliver amphiphilic, lipid-soluble, and water-soluble compounds to the gastrointestinal tract (112). Liposomes containing apigenin with a particle size of 103 nm were examined in a study using human colorectal cancer cell lines HCT-15 and HT-2 (99). The results demonstrated that apigenin liposomes improved anti-neoplastic activity in a tumor xenograft model and enhanced hemocompatibility and cytocompatibility with normal fibroblasts. Liposomal apigenin, which has high chemotherapeutic potential, can be injected intravenously. Apigenin encapsulated in solid lipid nanoparticle (SLNP) showed enhanced efficacy in the treatment of diabetes mellitus. This highly bioavailable AP-SLNP, which had a particle size of about 150 nm, demonstrated antioxidant and anti-inflammatory activities, decreasing NF-kB activity and increasing Nrf2 and HO-1 expression. It also has a protective effect against diabetes by reducing the amount of glucose in rat blood (100). Although it has been demonstrated that lipid-based carriers are appropriate for use as oral delivery systems, their circulation-longevity is decreased by significant gastrointestinal lipolysis (113). Additionally, studies have demonstrated the use of a non-water-quenching dye to restore lipids following lipolysis *in vivo*. It is possible to track the reconstitution of lipolytic products inside lipid-based carriers, which provides information on the health of the gastrointestinal system (114).

## 8. Application of apigenin in food

According to a large number of studies, the theoretical maximum daily intake of apigenin is approximately 50 mg, suggesting that it is a suitable dietary supplement (61). Apigenin has become a mature food additive and nutritional supplement with extensive application prospects. The effects of extreme gastrointestinal conditions and the external environment on the stability of apigenin can be effectively reduced, and the bioavailability of apigenin can be improved by the delivery systems described above. It is important to design delivery systems comprehensively before introducing them into commercial food product development (115). The raw material used should reach food grade, and its economy should be high. Delivery systems should not affect the physicochemical or sensory properties of the final product. Delivery systems must be extremely stable, such that the substance can be consumed and completely broken down in the human gut without degradation during processing, transportation, or storage. Capsulized apigenin should have better bioavailability. Current applications of apigenin are primarily to introduce it into health foods, functional drinks, and colorants because of its anti-fatigue, anti-aging, and anti-cancer properties.

### 8.1. Application of apigenin in the development of health foods

Apigenin has strong anti-diabetic and anti-inflammatory activities, as well as immunoregulatory properties, and has good application prospects in the development of health foods. For example, *in vivo* animal studies have revealed the positive effects of celery-based apigenin-rich diets (AIN-93G control diet supplemented with 10% w/w celery-based apigenin rich extracts (25 μM apigenin-equivalent)) on the modulation of the LPS-induced miR-155 levels in mouse lungs (116). Importantly, it was further found that *in vivo*, concentrations of apigenin of ~1 μM, found in serum of mice fed with the celery-based apigenin rich diets effectively restored TNF-α expression to confer immune-regulatory activity (116, 117). Future experiments are guarantee to evaluate the therapeutic as well as the preventive potential of this diet.

### 8.2. Application of apigenin in functional beverages

It is well known that deep formulations containing apigenin have been developed by more and more commercial companies for application in the research and development of various energy drinks, including composite fruit drinks, breakfast fortified drinks, and sports drinks. Apigenin energy drinks have anti-fatigue, cooling, and refreshing properties, because apigenin fights inflammation and OS after a short period of intense exercise (118).

### 8.3. Application of apigenin in food processing

Apigenin can be used as a food additive and colorant in the food processing industry. Importantly, apigenin has been assessed as safe and effective and its use as an ingredient in food has been approved (119, 120). As a natural pigment, apigenin can replace nitrites, ensure food safety, and can be used for corrosion prevention and coloring of biscuits, jellies, and meat products. Apigenin is also used as a coloring agent in pastries, ice-creams, and confectionary.

### 8.4. Application of apigenin in cosmetics

Apigenin strongly absorbs UVB rays (wavelengths between 280 and 320 nm), and can be used in sunscreen cosmetics. However, the absorption of apigenin in region A (wavelengths between 320 and 400 nm) is small, so low concentrations of apigenin can also be used as a skin darkening agent in tanning oils (121). Apigenin has been used in cosmetics as a pigment stabilizer at a recommended concentration of 1%. It can also be used in creams and in combination with vitamins C, B12, B6, and B1 and is usually mixed with plant essential oils such as chamomile, calendula, and almond oil. High concentrations of apigenin can inhibit the activity of melanocytes (122); can be added to sunscreen, face cream, essence, toner, facial masks, and other cosmetics; and can also be used in shampoos and conditioners.

Apigenin has strong antioxidant properties, a strong trapping capacity for various oxygen-containing free radicals and can prevent the oxidative degradation of oil (123). Apigenin also has anti-inflammatory activity and can prevent skin problems, such as bullous pemphigoid, keratosis, and incomplete keratosis. In addition, apigenin relieves itchy scalps and can be used in hair care products. Therefore, apigenin is increasingly used for its cosmetic efficacy.

## 9. Conclusion and perspectives

Apigenin can be used extensively in the food industry because of its abundant bioactivities, and its influence on stability should be considered during processing; its bioavailability, digestion, and absorption in the human body should also be investigated in the future. Additional animal and cell models should be used in the future to simulate and verify the outcomes of *in vitro* digestion models. Further studies investigating the upstream regulators or receptors are needed to better understand the mechanisms underlying the modulation of bioactivity. To effectively utilize the bioactivity of apigenin, scientists should conduct additional research and development on apigenin delivery systems, particularly Pickering emulsion and hydrogel delivery systems.

## Author contributions

PC: writing—original draft preparation. PC, FC, ZG, and JL: writing—review and editing. BZ: supervision and approval. All authors contributed significantly to the writing of the manuscript, read, and approved the manuscript for publication.

## Funding

This work was supported by Hubei Provincial Natural Science Foundation of China (2022CFB003) and the Open Project of Hubei KeyLaboratory of Wudang Local Chinese Medicine Research (Hubei University of Medicine) (WDCM2022006).

## Conflict of interest

The authors declare that the research was conducted in the absence of any commercial or financial relationships that could be construed as a potential conflict of interest.

## Publisher’s note

All claims expressed in this article are solely those of the authors and do not necessarily represent those of their affiliated organizations, or those of the publisher, the editors and the reviewers. Any product that may be evaluated in this article, or claim that may be made by its manufacturer, is not guaranteed or endorsed by the publisher.

## References

[1] Salehi B Venditti A Sharifi-Rad M Kręgiel D Sharifi-Rad J Durazzo A. The therapeutic potential of Apigenin. Int J Mol Sci. (2019) 20:1305. doi: 10.3390/ijms20061305, PMID: 30875872 PMC6472148

[2] Li B Hu Y Wu T Feng Y Jiang C Du H. Apigenin-oxymatrine binary co-amorphous mixture: enhanced solubility, bioavailability, and anti-inflammatory effect. Food Chem. (2022) 373:131485. doi: 10.1016/j.foodchem.2021.131485, PMID: 34740050

[3] Adel M Zahmatkeshan M Akbarzadeh A Rabiee N Ahmadi S Keyhanvar P. Chemotherapeutic effects of Apigenin in breast cancer: preclinical evidence and molecular mechanisms; enhanced bioavailability by nanoparticles. Biotechnol Rep (Amst). (2022) 34:e00730. doi: 10.1016/j.btre.2022.e00730 35686000 PMC9171451

[4] Kashyap P Shikha D Thakur M Aneja A. Functionality of apigenin as a potent antioxidant with emphasis on bioavailability, metabolism, action mechanism and *in vitro* and *in vivo* studies: a review. J Food Biochem. (2022) 46:e13950. doi: 10.1111/jfbc.13950, PMID: 34569073

[5] Hu Q Qu C Xiao X Zhang W Jiang Y Wu Z. Flavonoids on diabetic nephropathy: advances and therapeutic opportunities. Chin Med. (2021) 16:74. doi: 10.1186/s13020-021-00485-4, PMID: 34364389 PMC8349014

[6] Majma Sanaye P Mojaveri MR Ahmadian R Sabet Jahromi M Bahramsoltani R. Apigenin and its dermatological applications: a comprehensive review. Phytochemistry. (2022) 203:113390. doi: 10.1016/j.phytochem.2022.113390, PMID: 35998830

[7] Singh B Kumar A Malik AK. Flavonoids biosynthesis in plants and its further analysis by capillary electrophoresis. Electrophoresis. (2017) 38:820–32. doi: 10.1002/elps.201600334, PMID: 27921314

[8] Wang Y Liu XJ Chen JB Cao JP Li X Sun CD. Citrus flavonoids and their antioxidant evaluation. Crit Rev Food Sci Nutr. (2022) 62:3833–54. doi: 10.1080/10408398.2020.1870035 33435726

[9] Singh D Khan MA Siddique HR. Apigenin, a plant flavone playing Noble roles in Cancer prevention via modulation of key cell signaling networks. Recent Pat Anticancer Drug Discov. (2019) 14:298–311. doi: 10.2174/1574892814666191026095728, PMID: 31746310

[10] Sharma A Ghani A Sak K Tuli HS Sharma AK Setzer WN. Probing into therapeutic anti-cancer potential of Apigenin: recent trends and future directions. Recent Patents Inflamm Allergy Drug Discov. (2019) 13:124–33. doi: 10.2174/1872213X13666190816160240, PMID: 31418666

[11] Lee H Kim BG Kim M Ahn JH. Biosynthesis of two flavones, Apigenin and Genkwanin, in *Escherichia coli*. J Microbiol Biotechnol. (2015) 25:1442–8. doi: 10.4014/jmb.1503.03011, PMID: 25975614

[12] Marín L Gutiérrez-Del-Río I Yagüe P Manteca Á Villar CJ Lombó F. De novo biosynthesis of Apigenin, Luteolin, and Eriodictyol in the Actinomycete Streptomyces albus and production improvement by feeding and spore conditioning. Front Microbiol. (2017) 8:921. doi: 10.3389/fmicb.2017.00921, PMID: 28611737 PMC5447737

[13] Hutchins WA Wheeler TS. 17. Chalkones: a new synthesis of chrysin, apigenin, and luteolin. J Chem Soc. (1939):91–4. doi: 10.1039/jr9390000091

[14] Keumoe R Koffi JG Dize D Fokou PVT Tchamgoue J Ayong L. Identification of 3,3'-O-dimethylellagic acid and apigenin as the main antiplasmodial constituents of Endodesmia calophylloides Benth and Hymenostegia afzelii (Oliver.) harms. BMC Complement Med Ther. (2021) 21:3352. doi: 10.1186/s12906-021-03352-9 PMC8243547 34187456

[15] Süntar I Küpeli Akkol E Keles H Yesilada E Sarker SD. Exploration of the wound healing potential of Helichrysum graveolens (Bieb.) sweet: isolation of apigenin as an active component. J Ethnopharmacol. (2013) 149:103–10. doi: 10.1016/j.jep.2013.06.006, PMID: 23764736

[16] Che Zain MS Osman MF Lee SY Shaari K. UHPLC-UV/PDA method validation for simultaneous quantification of Luteolin and Apigenin derivatives from *Elaeis guineensis* leaf extracts: an application for antioxidant herbal preparation. Molecules. (2021) 26:1084. doi: 10.3390/molecules26041084, PMID: 33669484 PMC7922162

[17] Peng H Xing Y Gao L Zhang L Zhang G. Simultaneous separation of apigenin, luteolin and rosmarinic acid from the aerial parts of the copper-tolerant plant Elsholtzia splendens. Environ Sci Pollut Res Int. (2014) 21:8124–32. doi: 10.1007/s11356-014-2747-5, PMID: 24671394

[18] Yoshino Y Marunaka K Kobayashi M Matsunaga H Shu S Matsunaga T. Protective effects of ethanol extract of Brazilian green Propolis and Apigenin against weak ultraviolet ray-B-induced barrier dysfunction via suppressing nitric oxide production and Mislocalization of Claudin-1 in HaCaT cells. Int J Mol Sci. (2021) 22:10326. doi: 10.3390/ijms221910326, PMID: 34638666 PMC8508977

[19] Chiang YH Wu YT Lin LC Tsai TH. Comparative biotransformation of luteolin and apigenin from the flower extract and the stem-and-leaf extract of *Dendranthema morifolium* Ramat Tzvel. in rats. J Sci Food Agric. (2021) 101:4934–45. doi: 10.1002/jsfa.11137, PMID: 33543470

[20] Kawamura H Mishima K Sharmin T Ito S Kawakami R Kato T. Ultrasonically enhanced extraction of luteolin and apigenin from the leaves of *Perilla frutescens* (L.) Britt using liquid carbon dioxide and ethanol. Ultrason Sonochem. (2016) 29:19–26. doi: 10.1016/j.ultsonch.2015.08.016, PMID: 26584980

[21] Yang YC Wei MC. Development and characterization of a green procedure for apigenin extraction from Scutellaria barbata D. Don Food Chem. (2018) 252:381–9. doi: 10.1016/j.foodchem.2017.12.086, PMID: 29478557

[22] Zhang Q Zhou MM Chen PL Cao YY Tan XL. Optimization of ultrasonic-assisted enzymatic hydrolysis for the extraction of luteolin and apigenin from celery. J Food Sci. (2011) 76:C680–5. doi: 10.1111/j.1750-3841.2011.02174.x, PMID: 22417412

[23] Han D Row KH. Determination of luteolin and apigenin in celery using ultrasonic-assisted extraction based on aqueous solution of ionic liquid coupled with HPLC quantification. J Sci Food Agric. (2011) 91:2888–92. doi: 10.1002/jsfa.4553, PMID: 21748734

[24] Wang H Yang L Zu Y Zhao X. Microwave-assisted simultaneous extraction of luteolin and apigenin from tree peony pod and evaluation of its antioxidant activity. Sci World J. (2014) 2014:506971:1–12. doi: 10.1155/2014/506971 PMC4227382 25405227

[25] Nguyen Thu H Vu Thi Huyen T Nguyen VP. Application of multivariate linear regression models for selection of deep eutectic solvent for extraction of apigenin and luteolin from *Chrysanthemum indicum* L. Phytochem Anal. (2022) 33:427–40. doi: 10.1002/pca.3099 34808692

[26] Hostetler GL Ralston RA Schwartz SJ. Flavones: food sources, bioavailability, metabolism, and bioactivity. Adv Nutr. (2017) 8:423–35. doi: 10.3945/an.116.012948, PMID: 28507008 PMC5421117

[27] Zalai D Kopp J Kozma B Küchler M Herwig C Kager J. Microbial technologies for biotherapeutics production: key tools for advanced biopharmaceutical process development and control. Drug Discov Today Technol. (2020) 38:9–24. doi: 10.1016/j.ddtec.2021.04.001, PMID: 34895644

[28] Lefort ÉC Blay J. Apigenin and its impact on gastrointestinal cancers. Mol Nutr Food Res. (2013) 57:126–44. doi: 10.1002/mnfr.201200424, PMID: 23197449

[29] Pápay ZE Kállai-Szabó N Balogh E Ludányi K Klebovich I Antal I. Controlled release Oral delivery of Apigenin containing pellets with antioxidant activity. Curr Drug Deliv. (2017) 14:145–54. doi: 10.2174/1567201813666160602193047, PMID: 27264725

[30] Jakubczyk K Dec K Kałduńska J Kawczuga D Kochman J Janda K. Reactive oxygen species – sources, functions, oxidative damage. Pol Merkur Lekarski. (2020) 48:124–7. PMID: 32352946

[31] Di Meo S Venditti P. Evolution of the knowledge of free radicals and other oxidants. Oxidative Med Cell Longev. (2020) 2020:9829176. doi: 10.1155/2020/9829176 PMC7201853 32411336

[32] Cyr AR Huckaby LV Shiva SS Zuckerbraun BS. Nitric oxide and endothelial dysfunction. Crit Care Clin. (2020) 36:307–21. doi: 10.1016/j.ccc.2019.12.009, PMID: 32172815 PMC9015729

[33] Jang JY Sung B Kim ND. Role of induced programmed cell death in the Chemopreventive potential of Apigenin. Int J Mol Sci. (2022) 23:3757. doi: 10.3390/ijms23073757, PMID: 35409117 PMC8999072

[34] Thiruvengadam M Venkidasamy B Subramanian U Samynathan R Ali Shariati M Rebezov M. Bioactive compounds in oxidative stress-mediated diseases: targeting the NRF2/ARE signaling pathway and epigenetic regulation. Antioxidants (Basel). (2021) 10:1859. doi: 10.3390/antiox10121859, PMID: 34942962 PMC8698417

[35] Yi YS. Regulatory roles of flavonoids on Inflammasome activation during inflammatory responses. Mol Nutr Food Res. (2018) 62:e1800147. doi: 10.1002/mnfr.201800147, PMID: 29774640

[36] Al-Khayri JM Sahana GR Nagella P Joseph BV Alessa FM Al-Mssallem MQ. Flavonoids as potential anti-inflammatory molecules: a review. Molecules. (2022) 27:2901. doi: 10.3390/molecules27092901, PMID: 35566252 PMC9100260

[37] Park CH Min SY Yu HW Kim K Kim S Lee HJ. Effects of Apigenin on RBL-2H3, RAW264.7, and HaCaT cells: anti-allergic, anti-inflammatory, and skin-protective activities. Int J Mol Sci. (2020) 21:4620. doi: 10.3390/ijms21134620 32610574 PMC7370139

[38] Lee JH Zhou HY Cho SY Kim YS Lee YS Jeong CS. Anti-inflammatory mechanisms of apigenin: inhibition of cyclooxygenase-2 expression, adhesion of monocytes to human umbilical vein endothelial cells, and expression of cellular adhesion molecules. Arch Pharm Res. (2007) 30:1318–27. doi: 10.1007/BF02980273 18038911

[39] Patel M Singh S. Apigenin Attenuates Functional and Structural Alterations via Targeting NF-kB/Nrf2 Signaling Pathway in LPS-Induced Parkinsonism in Experimental Rats : Apigenin Attenuates LPS-Induced Parkinsonism in Experimental Rats. Neurotox Res. (2022) 40:941–60. doi: 10.1007/s12640-022-00521-7 35608813

[40] Al-Ghraiybah NF Wang J Alkhalifa AE Roberts AB Raj R Yang E. Glial cell-mediated Neuroinflammation in Alzheimer's disease. Int J Mol Sci. (2022) 23:10572. doi: 10.3390/ijms231810572, PMID: 36142483 PMC9502483

[41] Chen P Huo X Liu W Li K Sun Z Tian J. Apigenin exhibits anti-inflammatory effects in LPS-stimulated BV2 microglia through activating GSK3β/Nrf2 signaling pathway. Immunopharmacol Immunotoxicol. (2020) 42:9–16. doi: 10.1080/08923973.2019.1688345, PMID: 31760890

[42] Chumsakul O Wakayama K Tsuhako A Baba Y Takai Y Kurose T. Apigenin regulates activation of microglia and counteracts retinal degeneration. J Ocul Pharmacol Ther. (2020) 36:311–9. doi: 10.1089/jop.2019.0163, PMID: 32379991

[43] Coelho PLC Amparo JAO da Silva AB da Silva KC Braga-de-Souza S Barbosa PR. Apigenin from *Croton betulaster* Müll restores the immune profile of microglia against glioma cells. Phytother Res. (2019) 33:3191–202. doi: 10.1002/ptr.6491 31468624

[44] Ginwala R Bhavsar R Moore P Bernui M Singh N Bearoff F. Apigenin modulates dendritic cell activities and curbs inflammation via RelB inhibition in the context of Neuroinflammatory diseases. J Neuroimmune Pharmacol. (2021) 16:403–24. doi: 10.1007/s11481-020-09933-8, PMID: 32607691 PMC7772281

[45] Singh D Gupta M Sarwat M Siddique HR. Apigenin in cancer prevention and therapy: a systematic review and meta-analysis of animal models. Crit Rev Oncol Hematol. (2022) 176:103751. doi: 10.1016/j.critrevonc.2022.103751, PMID: 35752426

[46] Zhou Y Yu Y Lv H Zhang H Liang T Zhou G. Apigenin in cancer therapy: from mechanism of action to nano-therapeutic agent. Food Chem Toxicol. (2022) 168:113385. doi: 10.1016/j.fct.2022.113385, PMID: 36007853

[47] Tong J Shen Y Zhang Z Hu Y Zhang X Han L. Apigenin inhibits epithelial-mesenchymal transition of human colon cancer cells through NF-κB/snail signaling pathway. Biosci Rep. (2019) 39:452. doi: 10.1042/BSR20190452 PMC6522743 30967496

[48] Nelson N Szekeres K Iclozan C Rivera IO McGill A Johnson G. Apigenin: selective CK2 inhibitor increases Ikaros expression and improves T cell homeostasis and function in murine pancreatic cancer. PLoS One. (2017) 12:e0170197. doi: 10.1371/journal.pone.0170197, PMID: 28152014 PMC5289423

[49] Nicholas C Batra S Vargo MA Voss OH Gavrilin MA Wewers MD. Apigenin blocks lipopolysaccharide-induced lethality *in vivo* and proinflammatory cytokines expression by inactivating NF-kappaB through the suppression of p65 phosphorylation. J Immunol. (2007) 179:7121–7. doi: 10.4049/jimmunol.179.10.7121 17982104

[50] Zheng S Cao P Yin Z Wang X Chen Y Yu M. Apigenin protects mice against 3,5-diethoxycarbonyl-1,4-dihydrocollidine-induced cholestasis. Food Funct. (2021) 12:2323–34. doi: 10.1039/D0FO02910F, PMID: 33620063

[51] Chan LP Chou TH Ding HY Chen PR Chiang FY Kuo PL. Apigenin induces apoptosis via tumor necrosis factor receptor-and Bcl-2-mediated pathway and enhances susceptibility of head and neck squamous cell carcinoma to 5-fluorouracil and cisplatin. Biochim Biophys Acta. (2012) 1820:1081–91. doi: 10.1016/j.bbagen.2012.04.013, PMID: 22554915

[52] Hassannia B Vandenabeele P Vanden BT. Targeting Ferroptosis to Iron out Cancer. Cancer Cell. (2019) 35:830–49. doi: 10.1016/j.ccell.2019.04.002, PMID: 31105042

[53] Chen X Kang R Kroemer G Tang D. Ferroptosis in infection, inflammation, and immunity. J Exp Med. (2021) 218:e20210518. doi: 10.1084/jem.20210518, PMID: 33978684 PMC8126980

[54] Koppula P Zhuang L Gan B. Cystine transporter SLC7A11/xCT in cancer: ferroptosis, nutrient dependency, and cancer therapy. Protein Cell. (2021) 12:599–620. doi: 10.1007/s13238-020-00789-5, PMID: 33000412 PMC8310547

[55] Adham AN Abdelfatah S Naqishbandi AM Mahmoud N Efferth T. Cytotoxicity of apigenin toward multiple myeloma cell lines and suppression of iNOS and COX-2 expression in STAT1-transfected HEK293 cells. Phytomedicine. (2021) 80:153371. doi: 10.1016/j.phymed.2020.153371, PMID: 33070080

[56] Liu R Rong G Liu Y Huang W He D Lu R. Delivery of apigenin-loaded magnetic Fe2O3/Fe3O4@mSiO2 nanocomposites to A549 cells and their antitumor mechanism. Mater Sci Eng C Mater Biol Appl. (2021) 120:111719. doi: 10.1016/j.msec.2020.111719, PMID: 33545870

[57] Gangwar V Garg A Lomore K Korla K Bhat SS Rao RP. Immunomodulatory effects of a concoction of natural bioactive compounds-mechanistic insights. Biomedicine. (2021) 9:1522. doi: 10.3390/biomedicines9111522, PMID: 34829751 PMC8615223

[58] Wang X Li J Zhao D Li J. Therapeutic and preventive effects of apigenin in cerebral ischemia: a review. Food Funct. (2022) 13:11425–37. doi: 10.1039/D2FO02599J, PMID: 36314275

[59] Xu Y Li X Wang H. Protective roles of Apigenin against Cardiometabolic diseases: a systematic review. Front Nutr. (2022) 9:875826. doi: 10.3389/fnut.2022.875826, PMID: 35495935 PMC9051485

[60] Tang D Chen K Huang L Li J. Pharmacokinetic properties and drug interactions of apigenin, a natural flavone. Expert Opin Drug Metab Toxicol. (2017) 13:323–30. doi: 10.1080/17425255.2017.1251903, PMID: 27766890

[61] Wang M Firrman J Liu L Yam K. A review on flavonoid Apigenin: dietary intake, ADME, antimicrobial effects, and interactions with human gut microbiota. Biomed Res Int. (2019) 2019:1–18. doi: 10.1155/2019/7010467 PMC6817918 31737673

[62] Hanske L Loh G Sczesny S Blaut M Braune A. The bioavailability of apigenin-7-glucoside is influenced by human intestinal microbiota in rats. J Nutr. (2009) 139:1095–102. doi: 10.3945/jn.108.102814, PMID: 19403720

[63] Cardona F Andrés-Lacueva C Tulipani S Tinahones FJ Queipo-Ortuño MI. Benefits of polyphenols on gut microbiota and implications in human health. J Nutr Biochem. (2013) 24:1415–22. doi: 10.1016/j.jnutbio.2013.05.001, PMID: 23849454

[64] Liu Y Hu M. Absorption and metabolism of flavonoids in the caco-2 cell culture model and a perused rat intestinal model. Drug Metab Dispos. (2002) 30:370–7. doi: 10.1124/dmd.30.4.370, PMID: 11901089

[65] Zhang J Liu D Huang Y Gao Y Qian S. Biopharmaceutics classification and intestinal absorption study of apigenin. Int J Pharm. (2012) 436:311–7. doi: 10.1016/j.ijpharm.2012.07.002, PMID: 22796171

[66] Gradolatto A Basly JP Berges R Teyssier C Chagnon MC Siess MH. Pharmacokinetics and metabolism of apigenin in female and male rats after a single oral administration. Drug Metab Dispos. (2005) 33:49–54. doi: 10.1124/dmd.104.000893, PMID: 15466493

[67] Chen T Li LP Lu XY Jiang HD Zeng S. Absorption and excretion of luteolin and apigenin in rats after oral administration of *Chrysanthemum morifolium* extract. J Agric Food Chem. (2007) 55:273–7. doi: 10.1021/jf062088r 17227053

[68] Barr JT Tran TB Rock BM Wahlstrom JL Dahal UP. Strain-dependent variability of early discovery small molecule pharmacokinetics in mice: does strain matter? Drug Metab Dispos. (2020) 48:613–21. doi: 10.1124/dmd.120.090621, PMID: 32474442

[69] Chen J Lin H Hu M. Metabolism of flavonoids via enteric recycling: role of intestinal disposition. J Pharmacol Exp Ther. (2003) 304:1228–35. doi: 10.1124/jpet.102.046409, PMID: 12604700

[70] Michaelis M Rothweiler F Nerreter T Sharifi M Ghafourian T Cinatl J. Karanjin interferes with ABCB1, ABCC1, and ABCG2. J Pharm Pharm Sci. (2014) 17:92–105. doi: 10.18433/J3BW2S, PMID: 24735762

[71] Braidot E Zancani M Petrussa E Peresson C Bertolini A Patui S. Transport and accumulation of flavonoids in grapevine(*Vitis vinifera* L.). Plant Signal Behav. (2008) 3:626–32. doi: 10.4161/psb.3.9.6686, PMID: 19513253 PMC2634543

[72] Meyer H Bolarinwa A Wolfram G Linseisen J. Bioavailability of apigenin from apiin-rich parsley in humans. Ann Nutr Metabol. (2006) 50:167–72. doi: 10.1159/000090736, PMID: 16407641

[73] Gonzales GB Smagghe G Grootaert C Zotti M Raes K Van Camp J. Flavonoid interactions during digestion, absorption, distribution and metabolism: a sequential structure-activity/property relationship-based approach in the study of bioavailability and bioactivity. Drug Metab Rev. (2015) 47:175–90. doi: 10.3109/03602532.2014.1003649, PMID: 25633078

[74] Wan L Guo C Yu Q Li Y Wang X Wang X. Quantitative determination of apigenin and its metabolism in rat plasma after intravenous bolus administration by HPLC coupled with tandem mass spectrometry. J Chromatogr B Analyt Technol Biomed Life Sci. (2007) 855:286–9. doi: 10.1016/j.jchromb.2007.05.007, PMID: 17561454

[75] Catelli Rocha Torres L de Oliveira G Sartori A de Souza Silva AP Matias de Alencar S. Bioaccessibility and uptake/epithelial transport of vitamin E: discoveries and challenges of *in vitro* and *ex vivo* assays. Food Res Int. (2022) 162:112143. doi: 10.1016/j.foodres.2022.112143, PMID: 36461364

[76] Kashyap P Anand S Thakur A. Evaluation of antioxidant and antimicrobial activity of Rhododendron arboreum flowers extract. Int J Food Ferment Technol. (2017) 7:123–8. doi: 10.5958/2277-9396.2017.00013.7

[77] Zhang DY Zu YG Fu YJ Luo M Wang W Gu CB. Enzyme pretreatment and negative pressure cavitation extraction of genistein and apigenin from the roots of pigeon pea [*Cajanus cajan* (L.) Millsp.] and the evaluation of antioxidant activity[J]. Industrial Crops Products. (2012) 37:311–20. doi: 10.1016/j.indcrop.2011.12.026

[78] Ng SP Wong KY Zhang L Zuo Z Lin G. Evaluation of the first-pass glucuronidation of selected flavones in gut by Caco-2 monolayer model. J Pharm Pharm Sci. (2004) 8:1–9. PMID: 15946592

[79] Wang SW Kulkarni KH Tang L Wang JR Yin T Daidoji T. Disposition of flavonoids via enteric recycling: UDP-glucuronosyltransferase (UGT) 1As deficiency in Gunn rats is compensated by increases in UGT2Bs activities. J Pharmacol Exp Ther. (2009) 329:1023–31. doi: 10.1124/jpet.108.147371, PMID: 19264971 PMC2683779

[80] Liu WN Shi J Fu Y Zhao XH. The stability and activity changes of Apigenin and Luteolin in human cervical Cancer Hela cells in response to heat treatment and Fe2+/Cu2+ addition. Foods. (2019) 8:346. doi: 10.3390/foods8080346, PMID: 31416279 PMC6723879

[81] Sentandreu E Carbonell L Rodrigo D Carbonell JV. Pulsed electric fields versus thermal treatment: equivalent processes to obtain equally acceptable citrus juices. J Food Prot. (2006) 69:2016–8. doi: 10.4315/0362-028X-69.8.2016, PMID: 16924935

[82] Morales-de la Peña M Salvia-Trujillo L Rojas-Graü MA Martín-Belloso O. Changes on phenolic and carotenoid composition of high intensity pulsed electric field and thermally treated fruit juice-soymilk beverages during refrigerated storage. Food Chem. (2011) 129:982–90. doi: 10.1016/j.foodchem.2011.05.058, PMID: 25212327

[83] Chu YH Chang CL Hsu HF. Flavonoid content of several vegetables and their antioxidant activity[J]. J Sci Food Agric. (2000) 80:561–6. doi: 10.1002/(SICI)1097-0010(200004)80:5<561::AID-JSFA574>3.0.CO;2-#

[84] Hostetler GL Riedl KM Schwartz SJ. Effects of food formulation and thermal processing on flavones in celery and chamomile. Food Chem. (2013) 141:1406–11. doi: 10.1016/j.foodchem.2013.04.051, PMID: 23790931 PMC3850025

[85] Harbourne N Jacquier JC O'Riordan D. Optimisation of the extraction and processing conditions of chamomile (*Matricaria chamomilla* L.) for incorporation into a beverage. Food Chem. (2009) 115:15–9. doi: 10.1016/j.foodchem.2008.11.044

[86] Mullen W Marks SC Crozier A. Evaluation of phenolic compounds in commercial fruit juices and fruit drinks. J Agric Food Chem. (2007) 55:3148–57. doi: 10.1021/jf062970x, PMID: 17362029

[87] Sakakibara H Honda Y Nakagawa S Ashida H Kanazawa K. Simultaneous determination of all polyphenols in vegetables, fruits, and teas. J Agric Food Chem. (2003) 51:571–81. doi: 10.1021/jf020926l, PMID: 12537425

[88] Perez-Moral N Saha S Philo M Hart DJ Winterbone MS Hollands WJ. Comparative bio-accessibility, bioavailability and bioequivalence of quercetin, apigenin, glucoraphanin and carotenoids from freeze-dried vegetables incorporated into a baked snack versus minimally processed vegetables: evidence from in vitro models and a human bioavailability study. J Funct Foods. (2018) 48:410–9. doi: 10.1016/j.jff.2018.07.035, PMID: 30344649 PMC6189524

[89] McClements DJ Xiao H. Designing food structure and composition to enhance nutraceutical bioactivity to support cancer inhibition. Semin Cancer Biol. (2017) 46:215–26. doi: 10.1016/j.semcancer.2017.06.003, PMID: 28596014

[90] Meroni E Raikos V. Formulating orange oil-in-water beverage emulsions for effective delivery of bioactives: improvements in chemical stability, antioxidant activity and gastrointestinal fate of lycopene using carrier oils. Food Res Int. (2018) 106:439–45. doi: 10.1016/j.foodres.2018.01.013, PMID: 29579945

[91] Abcha I Souilem S Neves MA Wang Z Nefatti M Isoda H. Ethyl oleate food-grade O/W emulsions loaded with apigenin: insights to their formulation characteristics and physico-chemical stability. Food Res Int. (2019) 116:953–62. doi: 10.1016/j.foodres.2018.09.032, PMID: 30717028

[92] Kim BK Cho AR Park DJ. Enhancing oral bioavailability using preparations of apigenin-loaded W/O/W emulsions: *in vitro* and *in vivo* evaluations. Food Chem. (2016) 206:85–91. doi: 10.1016/j.foodchem.2016.03.052, PMID: 27041302

[93] Chou TH Nugroho DS Chang JY Cheng YS Liang CH Deng MJ. Encapsulation and characterization of Nanoemulsions based on an anti-oxidative polymeric Amphiphile for topical Apigenin delivery. Polymers (Basel). (2021) 13:1016. doi: 10.3390/polym13071016, PMID: 33806031 PMC8037426

[94] Jangdey MS Gupta A Saraf S. Fabrication, *in-vitro* characterization, and enhanced *in-vivo* evaluation of carbopol-based nanoemulsion gel of apigenin for UV-induced skin carcinoma. Drug Deliv. (2017) 24:1026–36. doi: 10.1080/10717544.2017.1344333, PMID: 28687053 PMC8241183

[95] Zhao X Wang Z. A pH-sensitive microemulsion-filled gellan gum hydrogel encapsulated apigenin: characterization and in vitro release kinetics. Colloids Surf B Biointerfaces. (2019) 178:245–52. doi: 10.1016/j.colsurfb.2019.03.015 30875583

[96] Ding B Chen H Wang C Zhai Y Zhai G. Preparation and in vitro evaluation of apigenin loaded lipid nanocapsules. J Nanosci Nanotechnol. (2013) 13:6546–52. doi: 10.1166/jnn.2013.7763, PMID: 24245113

[97] Zhai YJ Guo CY Hou JN Zhang WD Zhai GX. Preparation and *in vitro* characterization of apigemin-loaded nanostructured lipid carriers. Zhong Yao Cai. (2011) 34:962–5. PMID: 22017012

[98] Shukla R Kashaw SK Jain AP Lodhi S. Fabrication of Apigenin loaded gellan gum-chitosan hydrogels (GGCH-HGs) for effective diabetic wound healing. Int J Biol Macromol. (2016) 91:1110–9. doi: 10.1016/j.ijbiomac.2016.06.075, PMID: 27344952

[99] Banerjee K Banerjee S Mandal M. Enhanced chemotherapeutic efficacy of apigenin liposomes in colorectal cancer based on flavone-membrane interactions. J Colloid Interface Sci. (2017) 491:98–110. doi: 10.1016/j.jcis.2016.12.025, PMID: 28012918

[100] Li P Bukhari SNA Khan T Chitti R Bevoor DB Hiremath AR. Apigenin-loaded solid lipid nanoparticle attenuates diabetic nephropathy induced by Streptozotocin nicotinamide through Nrf2/HO-1/NF-kB Signalling pathway. Int J Nanomedicine. (2020) 15:9115–24. doi: 10.2147/IJN.S256494, PMID: 33244230 PMC7683501

[101] Verkempinck SHE Salvia-Trujillo L Moens LG Charleer L Van Loey AM Hendrickx ME. Emulsion stability during gastrointestinal conditions effects lipid digestion kinetics. Food Chem. (2018) 246:179–91. doi: 10.1016/j.foodchem.2017.11.001 29291837

[102] Elzayat A Adam-Cervera I Álvarez-Bermúdez O Muñoz-Espí R. Nanoemulsions for synthesis of biomedical nanocarriers. Colloids Surf B Biointerfaces. (2021) 203:111764. doi: 10.1016/j.colsurfb.2021.111764 33892282

[103] Ashaolu TJ. Nanoemulsions for health, food, and cosmetics: a review. Environ Chem Lett. (2021) 19:3381–95. doi: 10.1007/s10311-021-01216-9, PMID: 33746662 PMC7956871

[104] Gradzielski M Duvail M de Molina PM Simon M Talmon Y Zemb T. Using microemulsions: formulation based on knowledge of their Mesostructure. Chem Rev. (2021) 121:5671–740. doi: 10.1021/acs.chemrev.0c00812, PMID: 33955731

[105] Tapeinos C Battaglini M Ciofani G. Advances in the design of solid lipid nanoparticles and nanostructured lipid carriers for targeting brain diseases. J Control Release. (2017) 264:306–32. doi: 10.1016/j.jconrel.2017.08.033, PMID: 28844756 PMC6701993

[106] Patel P Patel M. Nanostructured lipid carriers-a versatile carrier for Oral delivery of lipophilic drugs. Recent Pat Nanotechnol. (2021) 15:154–64. doi: 10.2174/1872210514666200909154959, PMID: 32912129

[107] Ahmad J Rizwanullah M Amin S Warsi MH Ahmad MZ Barkat MA. Nanostructured lipid carriers (NLCs): nose-to-brain delivery and Theranostic application. Curr Drug Metab. (2020) 21:1136–43. doi: 10.2174/1389200221666200719003304, PMID: 32682366

[108] Dutta D Chakraborty A Mukherjee B Gupta S. Aptamer-conjugated Apigenin nanoparticles to target colorectal carcinoma: a promising safe alternative of colorectal Cancer chemotherapy. ACS Appl Bio Mater. (2018) 1:1538–56. doi: 10.1021/acsabm.8b00441, PMID: 34996205

[109] Oliva N Conde J Wang K Artzi N. Designing Hydrogels for On-Demand Therapy. Acc Chem Res. (2017) 50:669–79. doi: 10.1021/acs.accounts.6b00536, PMID: 28301139 PMC6527116

[110] Bernhard S Tibbitt MW. Supramolecular engineering of hydrogels for drug delivery. Adv Drug Deliv Rev. (2021) 171:240–56. doi: 10.1016/j.addr.2021.02.002 33561451

[111] Shah S Dhawan V Holm R Nagarsenker MS Perrie Y. Liposomes: advancements and innovation in the manufacturing process. Adv Drug Deliv Rev. (2020) 154-155:102–22. doi: 10.1016/j.addr.2020.07.002, PMID: 32650041

[112] Guimarães D Cavaco-Paulo A Nogueira E. Design of liposomes as drug delivery system for therapeutic applications. Int J Pharm. (2021) 601:120571. doi: 10.1016/j.ijpharm.2021.120571 33812967

[113] Thomas N Holm R Rades T Müllertz A. Characterising lipid lipolysis and its implication in lipid-based formulation development. AAPS J. (2012) 14:860–71. doi: 10.1208/s12248-012-9398-6, PMID: 22956477 PMC3475863

[114] Xia F Fan W Jiang S Ma Y Lu Y Qi J. Size-dependent translocation of Nanoemulsions via Oral delivery. ACS Appl Mater Interfaces. (2017) 9:21660–72. doi: 10.1021/acsami.7b04916, PMID: 28616962

[115] Li D Wei Z Xue C. Alginate-based delivery systems for food bioactive ingredients: an overview of recent advances and future trends. Compr Rev Food Sci Food Saf. (2021) 20:5345–69. doi: 10.1111/1541-4337.12840 34596328

[116] Arango D Diosa-Toro M Rojas-Hernandez LS Cooperstone JL Schwartz SJ Mo X. Dietary apigenin reduces LPS-induced expression of miR-155 restoring immune balance during inflammation. Mol Nutr Food Res. (2015) 59:763–72. doi: 10.1002/mnfr.201400705, PMID: 25641956 PMC7955240

[117] Hostetler GL Riedl KM Schwartz SJ. Endogenous enzymes, heat, and pH affect flavone profiles in parsley (*Petroselinum crispum* var. neapolitanum) and celery (*Apium graveolens*) during juice processing. J Agric Food Chem. (2012) 60:202–8. doi: 10.1021/jf2033736, PMID: 22224550 PMC3858576

[118] Harnly JM Doherty RF Beecher GR Holden JM Haytowitz DB Bhagwat S. Flavonoid content of U.S. fruits, vegetables, and nuts. J Agric Food Chem. (2006) 54:9966–77. doi: 10.1021/jf061478a, PMID: 17177529

[119] Song Y Manson JE Buring JE Sesso HD Liu S. Associations of dietary flavonoids with risk of type 2 diabetes, and markers of insulin resistance and systemic inflammation in women: a prospective study and cross-sectional analysis. J Am Coll Nutr. (2005) 24:376–84. doi: 10.1080/07315724.2005.10719488 16192263

[120] Nielsen SE Young JF Daneshvar B Lauridsen ST Knuthsen P Sandström B. Effect of parsley (*Petroselinum crispum*) intake on urinary apigenin excretion, blood antioxidant enzymes and biomarkers for oxidative stress in human subjects. Br J Nutr. (1999) 81:447–55. doi: 10.1017/S000711459900080X, PMID: 10615220

[121] Jangdey MS Kaur CD Saraf S. Efficacy of Concanavalin-a conjugated nanotransfersomal gel of apigenin for enhanced targeted delivery of UV induced skin malignant melanoma. Artif Cells Nanomed Biotechnol. (2019) 47:904–16. doi: 10.1080/21691401.2019.1578784, PMID: 30856018

[122] Zhang B Wang J Zhao G Lin M Lang Y Zhang D. Apigenin protects human melanocytes against oxidative damage by activation of the Nrf2 pathway. Cell Stress Chaperones. (2020) 25:277–85. doi: 10.1007/s12192-020-01071-7, PMID: 31953635 PMC7058778

[123] Vergani L Vecchione G Baldini F Grasselli E Voci A Portincasa P. Polyphenolic extract attenuates fatty acid-induced steatosis and oxidative stress in hepatic and endothelial cells. Eur J Nutr. (2018) 57:1793–805. doi: 10.1007/s00394-017-1464-5, PMID: 28526925

